# A coordinate-based meta-analysis of acupuncture for chronic pain: Evidence from fMRI studies

**DOI:** 10.3389/fnins.2022.1049887

**Published:** 2022-12-14

**Authors:** Zheng Yu, Rong-Rong Wang, Wei Wei, Li-Ying Liu, Chuan-Biao Wen, Shu-Guang Yu, Xiao-Li Guo, Jie Yang

**Affiliations:** ^1^School of Intelligent Medicine, Chengdu University of Traditional Chinese Medicine, Chengdu, China; ^2^Acupuncture and Tuina School, Chengdu University of Traditional Chinese Medicine, Chengdu, China; ^3^Traditional Chinese Medicine Department, Chengdu Xinan Gynecological Hospital, Chengdu, China

**Keywords:** chronic pain, acupuncture, activation likelihood estimation, fMRI, systematic review

## Abstract

**Background:**

Chronic pain (CP) patients tend to represent aberrant functional brain activity. Acupuncture is an effective clinical treatment for CP, and some fMRI studies were conducted to discover the alternation of brain regions after acupuncture therapy for CP. However, the heterogeneity of neuroimaging studies has prevented researchers from systematically generalizing the central mechanisms of acupuncture in the treatment of CP.

**Methods:**

We searched bibliographic databases, including PubMed, EMBASE, PsycINFO, Web of Science Core Collection, ScienceDirect, China Academic Journal Network Publishing Database, etc., and trials registration platforms (From inception to September 1^st^, 2022). Two independent researchers assessed the study's bias and quality. Furthermore, activation likelihood estimation (ALE) analysis was applied to explore aberrant brain functional activity and acupuncture's central mechanism for CP.

**Results:**

Totally 14 studies with 524 CP patients were included in the study. ALE analysis showed that CP patients presented with decreased ALFF/ReHo in the precuneus, posterior cingulate cortex, right inferior parietal lobule, right superior temporal gyrus, cingulate gyrus, superior frontal gyrus, left medial frontal gyrus including medial prefrontal gurus, left middle frontal gyrus.

**Conclusion:**

This ALE meta-analysis pointed out that acupuncture could modulate the default mode network, the frontoparietal network to treat CP. This provided a systematic summary of the neuroimage biomarker of acupuncture for the treatment of CP.

**Systematic review registration:**

PROSPERO, identifier: CRD42021239633.

## Introduction

Chronic pain (CP), one of the most common and long-standing neurological disease, persists affecting the health and quality of life of patients worldwide (Goldberg and McGee, 2011; Dureja et al., 2014; Dahlhamer et al., 2018). Neuroscience evidence pointed out that CP itself altered brain activities, including endogenous pain control, suggesting that controlling pain became increasingly difficult as the pain became chronic (Fine, 2011; Hasvik et al., 2022; Noori et al., 2022). The introduction of the biopsychosocial model of pain during the past decade stimulated the development of more therapeutically effective and cost-effective interdisciplinary CP management programs.

In 1998, the National Institutes of Health (NIH) first endorsed acupuncture for treating CP disorders (Ulett et al., 1998). And throughout the past few years, acupuncture as a complementary alternative therapy has gained increasing popularity in the treatment of CP, with a large number of clinical studies demonstrating its safety and efficacy (Manheimer et al., 2007; Yuan et al., 2016; Vickers et al., 2018; Berger et al., 2021; Turkistani et al., 2021).

Resting state-functional magnetic resonance (rs-fMRI), an imaging technique based on the assessment of hemodynamic blood oxygen level-dependent (BOLD) effects, is frequently utilized to explore the brain modification of acupuncture for CP. For instance, fMRI studies have demonstrated that acupuncture could achieve therapeutic effects by modulating a variety of brain networks in CP, such as the emotional response network (Kong et al., 2002; Liu et al., 2021), the default network (DMN) (Hou et al., 2014; Zou et al., 2019b; Liu et al., 2020), the frontoparietal network (FPN) (Kong et al., 2013), etc.

Individual experiments with limited sample sizes and low test thresholds were, nevertheless, susceptible to yielding false positive results. In addition to the variety of experimental designs employed in the study, these factors provided substantial variation in the reported outcomes. Therefore, a synthesis of results across experiments is needed to determine consistent and systematic brian modulation mechanisms of acupuncture for CP. Activation likelihood estimation (ALE) is a reliable meta-analysis method based on whole-brain coordinates established by Turkeltaub et al. (2002, 2012), aiming at determining above-chance convergence of activation probabilities between experiments. Although ALE has been widely utilized in the field of neuroimaging (Chen et al., 2018; Chavanne and Robinson, 2021; Xu et al., 2021), no researchers have used ALE algorithm to examine the modulation of whole-brain function changes by acupuncture in CP patients.

To address the abovementioned issues, the purpose of this study was to systematically evaluate and analyze the changes of brain functional activity in CP patients and the regulation of brain functional activity after acupuncture treatment using a meta-analytical approach based on ALE algorithm. Our results may provide a more illustrative visual basis for elucidating the underlying neural mechanisms of acupuncture therapy for CP.

## Methods

### Literature search and study selection

#### Retrieval strategies

This systematic and standardized meta-analyses was corresponding to the Preferred Reporting Items (PRISMA) for sources including bibliographic databases, reference lists of eligible studies and review articles, and trial registers (Page et al., 2021). Bibliographic databases included MEDLINE *via* PubMed, EMBASE, PsycINFO, Web of Science Core Collection, ScienceDirect, China Academic Journal Network Publishing Database, China Doctoral Dissertation Full-text Database, China Excellent Master Dissertation Full-Text Database, Wanfang Database, Database of Chinese Sci-Tech Journals, and China Biomedical Literature Database. Trials register platforms included ClinicalTrials.gov, World Health Organization International Clinical Trials Registry Platform, Cochrane Central Register of Controlled Trials, and the Chinese Clinical Trial Register. Search date was from inception to September 1st, 2022. We only included studies published in Chinese or English. The search strategy of PubMed was shown in Supplementary material.

#### Selection criteria

##### Inclusion criteria

Each article was subsequently reviewed (first by abstract, then by full-text) for relevance to the study and inclusion of all following criteria:

1) Adults diagnosed as CP (Treede et al., 2015, 2019) (musculoskeletal, osteoarthritis, and headache, diagnosed using any recognized diagnostic criteria);
2) Administered acupuncture (defined as inserting the needle into the skin surface of the acupoint, such as manual acupuncture, electroacupuncture, etc.) as a therapeutic measure;
3) Were rs-fMRI studies;
4) Whole-brain analysis that reported coordinates for brain activities with standard anatomical reference space (Talairach or MNI);
5) Performed a statistical comparison (patients before and after acupuncture therapy or patients vs. healthy people).

##### Exclusion criteria

The studies met one of the following items were excluded:

1) Based on partial coverage or employing only region-of-interest (ROI) analyses;
2) The subjects (or a subgroup of subjects) were included in another study;
3) Studies based on ROI analyses or non-fMRI studies;
4) Were reviews or meta-analysis;
5) Incomplete information or secondary processed studies.

#### Data extraction

Two reviewers (WRR and WW) independently selected, extracted, and checked the data. The items included: author name, published year, title, journal name, CP categories, acupuncture type, sample size, gender differences, patients' age, analysis methods, foci details etc. When there was any disagreement, a third reviewer (YZ) participated in the decision.

### Quality assessments

Two reviewers (WRR and WW) scored the completeness using a 10-point checklist (Strakowski et al., 2003), and assessed the methodological quality using the Cochrane risk of bias (ROB) tools (https://training.cochrane.org/handbook). The measurement items of ROB tools contained seven different items: random sequence generation, allocation concealment, blinding of participants and personnel, blind of outcome assessment, incomplete outcome data and selective reporting and other sources of bias.

### ALE analysis

Ginger ALE software (http://www.brainmap.org/ale) was used for coordinate based meta-analysis. The differences in coordinate-based brain activity changes between CP and health control (HC), or post-pre-acupuncture in CP patients were assessed in this review. A cluster-level family wise error (FWE) correction was applied (*P* < 0.05) with thresholding permutations of 5,000 times (Müller et al., 2018). The resulting peak coordinates are reported in Talairach space. Mricron (available at www.mricro.com) was applied to brain visualization for results of ALE analysis. We did not perform the sensitivity analysis because of the small number of included studies.

## Results

### Search flow

**Figure 1:**
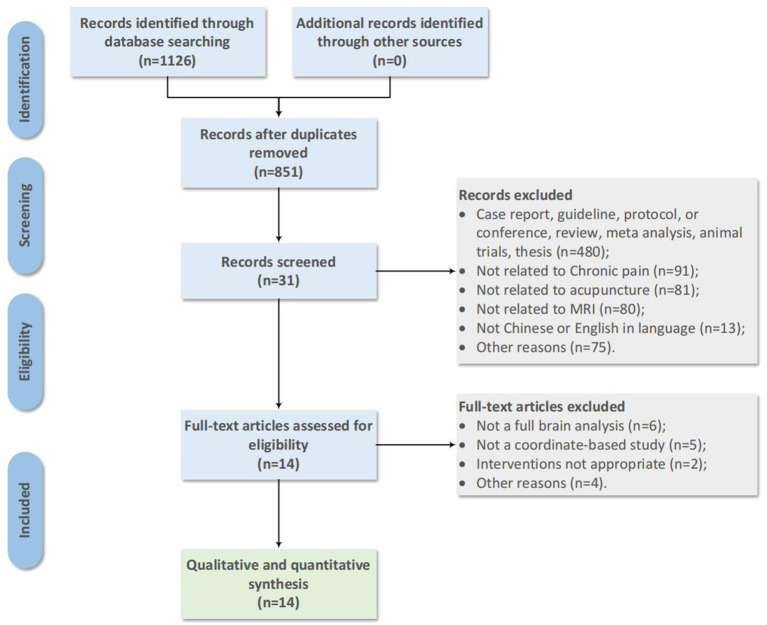
Flow diagram of literature search.

A total of 1,126 articles were retrieved. After excluding duplicate papers, a total of 851 articles were retained. Based on the title and abstract, a total of 820 papers were excluded, leaving 31 papers remaining. Following a comprehensive full-text scanning and application of established inclusion and exclusion criteria, 14 studies with 524 CP patients were included in the study (see Figure 1).

### Study characteristics

The included CP conditions were migraine without aura (MwoA) (Zhao et al., 2014; Han et al., 2017; Li et al., 2020; Ning et al., 2020; Jia et al., 2021; Liu et al., 2021); menstrual migraine without aura (MMoa) (Zhang et al., 2021); chronic neck pain (CNP) (Hou et al., 2014; Chen et al., 2015); chronic shoulder pain (CSP) (Zhang et al., 2018); chronic low back pain (cLBP) (Makary et al., 2018; Zou et al., 2019a; Liu et al., 2020); chronic knee osteoarthritis (KOA) (Qu et al., 2021). The studies using amplitude of low-frequency fluctuations (ALFF) or regional homogeneity (ReHo) as fMRI analysis methods were included in this study. The characteristics of included studies are summarized in Table 1.

**Table 1:** Characteristics and clinical information of the included studies.

**Source**	**Diagnosed**	**Sample size (*n*)**	**Gender (male/female)**	**Age (y)**	**Type of acupuncture**	**Foci (*n*)**	**Analysis method**	**Threshold**
Zhao et al. (2014)	MwoA	PCs: VA1 (20), VA2 (20)	VA1 (6/14), VA2 (8/12)	VA1: 32.9 ± 10.99; VA2: 37.25 ± 9.68	MA	VA_up: 21 VA_down: 15	ReHo	*P* < 0.05 (FDR)
Hou et al. (2014)	CNP	PCs: VA1 (25), VA2 (24)	VA1 (7/18), VA2 (12/12)	VA1: 24.73 ± 1.46; VA2: 24.79 ± 1.55	EA	VA_up: 4 VA_down: 3	ReHo	*NA*
		HCs: 19	HCs (8/11)	HCs: 25.58 ± 3.32	-	PCs_HCs_up: 0 PCs_HCs_down: 1		
Chen et al. (2015)	CNP	PCs: VA1 (25), VA2 (24)	VA1 (7/18), VA2 (14/10)	VA1: 25 ± 1; VA2: 25 ± 2	EA	VA_up: 0 VA_down: 4	ReHo	*P* < 0.01
		HCs: 19	HCs (8/11)	HCs: 25 ± 3	-	PCs_HCs_up: 0 PCs_HCs_down: 1		
Han et al. (2017)	MwoA	PCs: 10	PCs (2/8)	PCs: 31.7	MA	VA_up: 0 VA_down: 3	ReHo	*P* < 0.05
		HCs: 10	HCs (2/8)	*NA*	-	PCs_HCs_up: 10 PCs_HCs_down: 5		
Zhang et al. (2018)	CSP	PCs: VA1 (12), VA2 (8)	VA1 (6/6), VA2 (4/4)	VA1: 53.33 ± 5.26; VA2: 54.13 ± 7.45	MA	VA_up: 1 VA_down: 3	ReHo	*P* < 0.05
Makary et al. (2018)	cLBP	PCs: VA (28), PA (19)	VA (16/12), PA (9/10)	VA: 38.7 ± 13.1; PA: 39.5 ± 13.7	MA	VA_up: 65 VA_down: 36	fMRI signal	*NA*
Zou et al. (2019b)	cLBP	PCs: VA1 (16), VA2 (16)	VA1 (8/8), VA2 (7/9)	VA1: 48.53 ± 13.21; VA2: 44.21 ± 8.96	EA	*NA*	ReHo	*P* < 0.05
		HCs: 25	HCs (12/13)	PCs: 40.01 ± 9.75	-	PCs_HCs_up: 11 PCs_HCs_down: 11		
Liu et al. (2020)	cLBP	PCs: 12	PCs (6/6)	PCs: 61.42 ± 14.84	MA	VA_up: 0 VA_down: 1	ReHo/FC	*P* < 0.05 (FWE)
Ning et al. (2020)	MwoA	PCs: 19	PCs (3/16)	PCs: 28.23 ± 6.16	MA	VA_up: 1 VA_down: 4	ALFF	*P* < 0.05
		HCs: 18	PCs (4/14)	PCs: 27.16 ± 5.23	-	PCs_HCs_up: 3 PCs_HCs_down: 2		
Li et al. (2020)	MwoA	PCs: VA1 (12), VA2 (13), VA3 (14), SA (13), WT (18).	VA1 (2/10), VA2 (4/9), VA3 (2/12), SA (2/11), WT (4/14)	VA1:21.75 (20.70, 22.80); VA2:20.85 (19.57, 22.12); VA3: 20.78 (19.74, 21.83); SA: 21.38 (20.75, 22.02); WT: 21.61 (20.54, 22.68)	MA	*NA*	fALFF	*P* < 0.05 (FWE)
		HCs: 43	HCs (9/34)	HCs: 21.23 (20.96, 21.51)	-	PCs_HCs_up: 0 PCs_HCs_down: 4		
Zhang et al. (2021)	MMoa	PCs: VA (24), SA (20)	VA (0/24), SA (0/20)	VA (33.04 ± 6.43); SA (35.30 ± 9.43)	MA	VA_up: 4 VA_down: 3	ALFF/ReHo	*P* < 0.05
Qu et al. (2021)	KOA	PCs: 80	PCs (28/52)	PCs: 52.35 ± 4.62	MA	VA_up: 4 VA_down: 1	ALFF	*P* < 0.05
Liu et al. (2021)	MwoA	PCs: 37	PCs (6/31)	PCs: 37.97 ± 9.82	EA	VA_up: 1 VA_down: 0	ReHo	*P* < 0.05 (FWE)
		HCs: 15	HCs (2/13)	HCs: 34.88 ± 6.66	-	PCs_HCs_up: 0 PCs_HCs_down: 2		
Jia et al. (2021)	MwoA	PCs: 15	PCs (5/10)	PCs: 39.3 ± 12.1	MA	VA_up: 0 VA_down: 1	ReHo	*P* < 0.05

MwoA, migraine without aura; MMoa, menstrual migraine without aura; CNP, chronic neck pain; CSP, chronic shoulder pain; cLBP, chronic low back pain; KOA, chronic knee osteoarthritis; PCs, patient control; HCs, health controls; ReHo, regional homogeneity; ALFF, amplitude of low-frequency fluctuations; MA, manual acupuncture; EA, electroacupuncture; VA, verum acupuncture; PA, phantom acupuncture; SA, sham acupuncture; WT, waiting-list; FDR, false discovery rate; FWE, family wise error; NA, not available.

### Quality assessment

**Figure 2:**
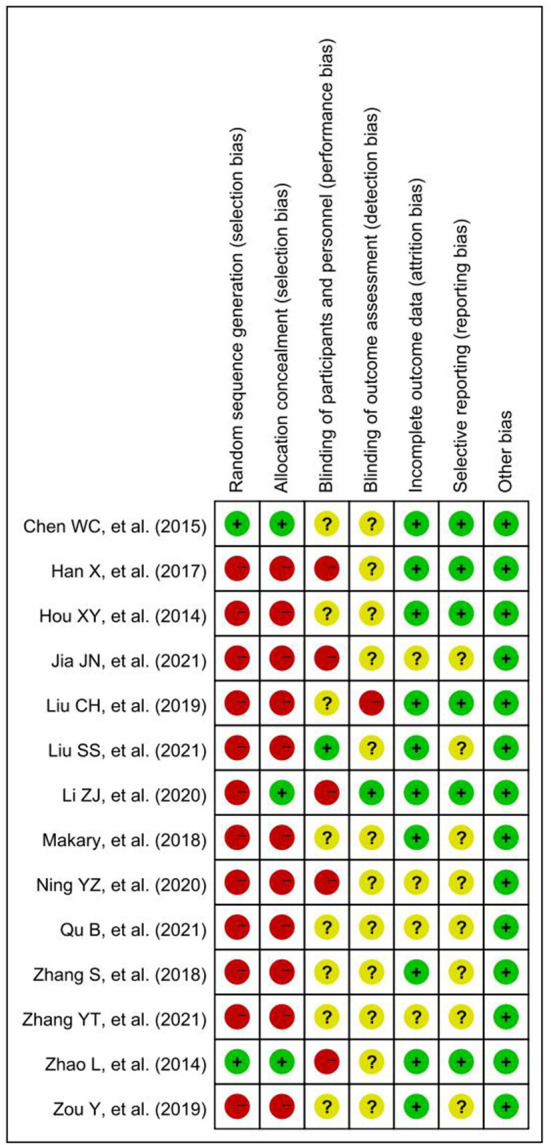
Risk of bias of each included study using Revman.

The quality control assessments by Strakowski's checklist showed that the completeness scores of the included studies are generally high (see Supplementary Table 1). Furthermore, two reviewers (WRR and WW) independently evaluated the methodological quality of the 14 included studies (see Figure 2 and Supplementary Figure 1). Only two studies (Zhao et al., 2014; Chen et al., 2015) reported entirely random sequences and allocation concealment, and only one research (Liu et al., 2021) mentioned participant blinding, which indicates that the majority of publications have a moderate risk of bias, predominantly in the areas of selection and performance bias.

### ALE results

Compared to the HC, patients with CP had decreased ALFF/ReHo of left caudate and left thalamus; increased ALFF/ReHo of right fusiform gyrus, left superior frontal gyrus (SFG) and bilateral medial frontal gyrus, left rectus, left cingulate cortex including posterior cingulate cortex (PCC) (see Supplementary Table 2, Supplementary Figures 2, 3).

**Figure 3:**
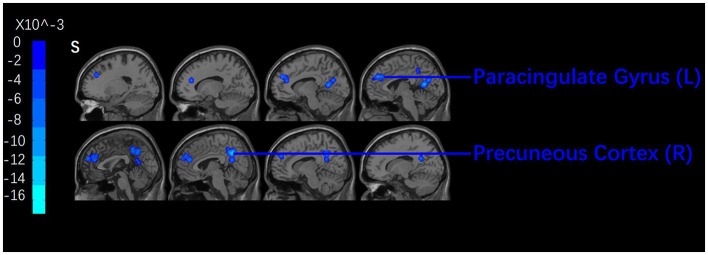
Brian regions with decreased signals pre- and post-acupuncture. A cluster-level FWE correction was applied (*P* < 0.05).

After acupuncture therapy, CP patients presented with decreased ALFF/ReHo in the precuneus, PCC, right inferior parietal lobule, right superior temporal gyrus (STG), cingulate gyrus, SFG, left medial frontal gyrus including medial prefrontal gurus (mPFC), left middle frontal gyrus (MFG) (see Figure 3 and Table 2).

**Table 2:** The results pf peak coordinates from ALE analysis pre- and post- acupuncture.

**Cluster #**	**x**	**y**	**z**	**ALE**	***P***	**Z**	**Label (nearest gray matter within 5 mm)**
1	4	−54	36	0.017405074	0.000	−5.037	Right Cerebrum.Parietal Lobe.Precuneus.Gray Matter.Brodmann area 7
1	−8	−58	12	0.012505891	0.000	−4.055	Left Cerebrum.Limbic Lobe.Posterior Cingulate.Gray Matter.Brodmann area 23
1	50	−64	36	0.009757249	0.000	−3.583	Right Cerebrum.Parietal Lobe.Inferior Parietal Lobule.Gray Matter.Brodmann area 39
1	26	−56	34	0.009065375	0.000	−3.458	No Gray Matter found
1	58	−60	26	0.008594425	0.000	−3.329	Right Cerebrum.Temporal Lobe.Superior Temporal Gyrus.Gray Matter.Brodmann area 39
1	42	−54	36	0.008330258	0.001	−3.244	Right Cerebrum.Parietal Lobe.Inferior Parietal Lobule.Gray Matter.Brodmann area 40
1	54	−52	46	0.008204443	0.001	−3.189	Right Cerebrum.Parietal Lobe.Inferior Parietal Lobule.Gray Matter.Brodmann area 40
1	−2	−44	46	0.008156347	0.001	−3.161	Left Cerebrum.Parietal Lobe.Precuneus.Gray Matter.Brodmann area 7
1	14	−52	24	0.007928049	0.001	−3.074	Right Cerebrum.Limbic Lobe.Cingulate Gyrus.Gray Matter.Brodmann area 31
1	−8	−68	22	0.007889439	0.001	−3.009	Left Cerebrum.Occipital Lobe.Precuneus.Gray Matter.Brodmann area 31
1	−2	−46	40	0.007888975	0.001	−3.009	Left Cerebrum.Limbic Lobe.Cingulate Gyrus.Gray Matter.Brodmann area 31
1	4	−54	22	0.006890667	0.004	−2.693	Right Cerebrum.Limbic Lobe.Posterior Cingulate.Gray Matter.Brodmann area 31
2	−6	40	30	0.009418508	0.000	−3.527	Left Cerebrum.Frontal Lobe.Medial Frontal Gyrus.Gray Matter.Brodmann area 9
2	−2	58	24	0.009083679	0.000	−3.462	Left Cerebrum.Frontal Lobe.Superior Frontal Gyrus.Gray Matter.Brodmann area 9
2	−8	48	30	0.009039057	0.000	−3.422	Left Cerebrum.Frontal Lobe.Superior Frontal Gyrus.Gray Matter.Brodmann area 9
2	4	50	30	0.008590198	0.000	−3.329	Right Cerebrum.Frontal Lobe.Superior Frontal Gyrus.Gray Matter.Brodmann area 9
2	−14	40	22	0.008063627	0.001	−3.125	Left Cerebrum.Frontal Lobe.Medial Frontal Gyrus.Gray Matter.Brodmann area 9
2	0	42	22	0.007801687	0.001	−2.988	Left Cerebrum.Frontal Lobe.Medial Frontal Gyrus.Gray Matter.Brodmann area 9
2	−36	32	36	0.006627638	0.004	−2.665	Left Cerebrum.Frontal Lobe.Middle Frontal Gyrus.Gray Matter.Brodmann area 9
2	−24	38	34	0.006448573	0.004	−2.634	Left Cerebrum.Frontal Lobe.Middle Frontal Gyrus.Gray Matter.Brodmann area 9

A negative Z value means decreased fMRI signal after acupuncture.

## Discussion

The current ALE meta-analysis pooled 14 fMRI studies based on coordinates encompassing 6 types of CP (MwoA, Mmoa, CNP, CSP, cLBP, KOA) to determine the effects of acupuncture on brain regions. The quality of the included studies were relatively moderate, as determined by the ROB checklist and quality reporting standard guidelines. According to rs-fMRI studies of CP patients treated with acupuncture, the modulation pattern of CP by acupuncture included a reduction of ALFF/ReHo signals in the DMN (precuneus, PCC), and FPN (SFG, mPFC, MFG). The findings confirmed that acupuncture may produce the therapeutic effect on CP by modulating the brain regions associated with emotion and cognition. In addition, similar to the findings of previous studies, abnormalities in the caudate, thalamus, fusiform gyrus, superior frontal gyrus, and medial prefrontal cortex were also identified in CP patients in this review. These abnormalities in CP were associated with pain processing, cognitive abnormalities, and emotion regulation functions.

The trajectory of chronic pain occurred over time, with intensity continuing and fluctuating (Mayr et al., 2022). Meanwhile, the perception of pain is a personal and multidimensional experience, with causes mood changes such as anxiety, depression and fear (Moriarty et al., 2011; Bushnell et al., 2013; Moseley and Vlaeyen, 2015). The present review partially revealed the abnormal functional activity in brain regions such as caudate, thalamus, and PFC in patients with CP. In fact, early studies already found abnormal functional activities in brain regions and networks, such as DMN and the salience network (Greenwald and Shafritz, 2018). At the cellular level, central sensitization may be reversed by degrading glutamate receptor pathways, but occurs rarely (Woolf, 2011). Instead, cortical brain regions may have top-down modulation to alleviate pain.

These results aligned with the previous ALE research (Ha et al., 2022) for acupuncture modifying musculoskeletal pain, in that both found therapeutic effects of acupuncture in the brain regions of the caudate, and thalamus. Methodological factors may have contributed to the differences between our study and those of earlier findings. Firstly, we included several kinds of CPs, including osteoarthritis, and headache, rather than just musculoskeletal pain. Secondly, we only included research reporting whole-brain analyses; some ROI-based approaches were omitted in order to eliminate the possibility of regional bias. We ultimately included 14 studies of moderate quality. Notably, the fact that almost all studies did not satisfy the recommended research a voxel-level threshold of *P* < 0.001 or a cluster-corrected threshold of *P* < 0.05 contributed to the drop in study quality. We also did not require such a test threshold in this study, as this may not have included enough studies.

Six different types of CP were covered in this study. Nevertheless, the mechanisms of acupuncture modulation of different type of CP might be distinct. For an instance, MwoA is neuropathic pain, whereas KOA is nociceptive pain. The pathogenic causes and mental state of the patients are diverse between these two disorders, resulting in modifications to distinct brain regions. In KOA, altered CNS activity led to a sense of pain in the absence of peripheral tissue injury or inflammation. Patients' anticipatory of pain may modify the forthcoming pain-evoking activation and pain sensation, as evidenced by limbic system brain activation (Jones et al., 2012). Nociceptive pain is somatic and visceral, which differ in their psychophysical and neurobiological mechanisms (Cervero, 2009). The main difference between them was the process of pain signaling and processing, which leads to different pain perception.

The ALE analysis in this study indicated the brain networks that acupuncture could modulate in CP patients were the DMN, FPN, suggesting a modulatory effect of acupuncture on CP at the neural network level. The DMN is an important resting brain functional network consisting of the precuneus, mPFC, inferior parietal lobule and PCC (Buckner et al., 2008; Raichle, 2010). As a major component of the DMN, the precuneus is responsible for processing negative emotions caused by pain or other discomfort and plays an important role in the cognitive function network (Cavanna and Trimble, 2006; Blessing et al., 2016). Our previous study discovered that acupuncture increased the unusually low precuneus brain metabolism in migraine sufferers (Yang et al., 2012). In conjunction with the results of ALE, it showed that acupuncture could modulate the abnormal precuneus function in CP to achieve a smooth strip of emotional cognition.

The cingulate gyrus is part of the DMN and limbic system, involving in pain perception and pain signaling. Patients with persistent neck and shoulder discomfort exhibited aberrant ALFF signal in the cingulate gyrus and precentral gyrus, compared to HCs (Yue and Du, 2020). Also, acupuncture could modulate brain regions involved in limbic system to achieve pain relief or relieve negative effects (Shi et al., 2020), such as hippocampus, parahippocampal gyrus, and anterior cingulate gyrus (Hui et al., 2010). In other words, acupuncture could modulate the abnormal functional activity of the limbic system in patients with CP.

The FPN was essential for pain processing involved in attention and cognitive control, especially the dorsolateral prefrontal cortex (DLPFC) and mPFC. Correspondingly, the present review suggested that acupuncture could modulate a wide range of FPN regions, including SFG, mPFC, MFG. Acupuncture has been extensively explored for its capacity to alleviate pain by modifying the dysfunctional DLPFC (He et al., 2022; Liu et al., 2022; Zhang et al., 2022). Consequencely, Ong's team revealed the importance of PFC during placebo analgesia and in establishing a connection between pain and pain alleviation in bouts of cognitive performance, anxiety, and cognitive decline (Ong et al., 2019). However, a PET-CT study revealed real acupuncture for KOA leaded to greater activation of the right DLPFC, ACC, and midbrain (Pariente et al., 2005) than sham acupuncture, indicating the differences in the modulation of brain regions by real and sham acupuncture.

## Limitations

This meta-analysis focused on whole-brain, resting brain function alterations in chronic pain improvement with acupuncture. Due to the general quality of the included studies, the results should be interpreted with caution. However, we included only 14 studies and did not perform a sensitivity analysis. The findings should be interpreted with caution due to the small number of included studies and the heterogeneity in trial design and demographic baseline between studies.

## Conclusion

This is the first cross-study coordinate-based meta-analysis to uncover the modulation mechanisms of acupuncture in CP. The results of this review provide neuroimaging evidence for acupuncture in the treatment of CP.

## Data availability statement

The original contributions presented in the study are included in the article/Supplementary material, further inquiries can be directed to the corresponding author/s.

## Author contributions

ZY, R-RW, WW, and L-YL: concept and design. ZY, R-RW, and WW: acquisition of data. ZY, JY, L-YL, and X-LG: drafting of the manuscript. S-GY and C-BW: critical revision of the manuscript. All authors contributed to the article and approved the submitted version.
