# Supplementary material for: A coordinate-based meta-analysis of acupuncture for chronic pain: Evidence from fMRI studies
Source: Front Neurosci. 2022 Dec 14;16:1049887. doi: 10.3389/fnins.2022.1049887 (PMC9795831; doi:10.3389/fnins.2022.1049887)
Supplement: Supplementary file 1 [file Data_Sheet_1.docx]

**Search strategy for PubMed**

**Search Date:** 2022.09.01

(((((((((MRI) OR (fMRI)) OR (Magnetic Resonance Imaging)) OR (Functional Magnetic Resonance Imaging)) OR (Functional MRI)) OR (BOLD)) OR (Blood oxygenation level dependent)) OR (rs-fMRI)) OR (resting-state fMRI)) AND ((((((Acupuncture) OR (Electroacupuncture)) OR (Acupuncture therapy)) OR (Auriculotherapy)) OR (Electric stimulation therapy)) OR (Acupuncture points)) AND (((((((((((((((((((((((((((((((((Osteoarthrosis[MeSH Terms]) OR (Osteoarthrosis[Title/Abstract])) OR

(chronic knee osteoarthritis[Title/Abstract])) OR (Arthrosis[Title/Abstract])) OR (Osteoarthritis[Title/Abstract])) OR (KOA[Title/Abstract])) OR (OA[Title/Abstract])) OR (Osteoarthritis-related knee pain[Title/Abstract])) OR (Knee pain[Title/Abstract]))OR (Headaches[MeSH Terms])) OR (tension-type headache[Title/Abstract])) OR (migraine[Title/Abstract])) OR (chronic headache[Title/Abstract])) OR (Cephalgia[Title/Abstract])) OR (Headaches[Title/Abstract]) OR (MwA[Title/Abstract])) OR (migraine with aura[Title/Abstract])) OR (MwoA[Title/Abstract])) OR (migraine without aura[Title/Abstract])) OR (Musculoskeletal Pain[MeSH Terms]) OR (Musculoskeletal Pain[Title/Abstract])) OR (Musculoskeletal Diseases[Title/Abstract])) OR (Muscle Disorder[Title/Abstract])) OR (Myofascial Pain[Title/Abstract])) OR (Muscular Diseases[Title/Abstract])) OR (chronic pain[MeSH Terms])) OR (chronic pain[Title/Abstract])) OR (Widespread Chronic Pain[Title/Abstract])) OR (Shoulder Pain[MeSH Terms]) OR (Shoulder Pain[Title/Abstract])) OR (Back Pain [MeSH Terms]) OR (Low Back Pain[Title/Abstract])) OR (Lumbago[Title/Abstract])) OR (Backache[Title/Abstract])) OR (Back Pain[Title/Abstract])) OR (Cervicalgia[MeSH Terms]) OR (Neck pain[Title/Abstract])) OR (Cervicalgia[Title/Abstract]))


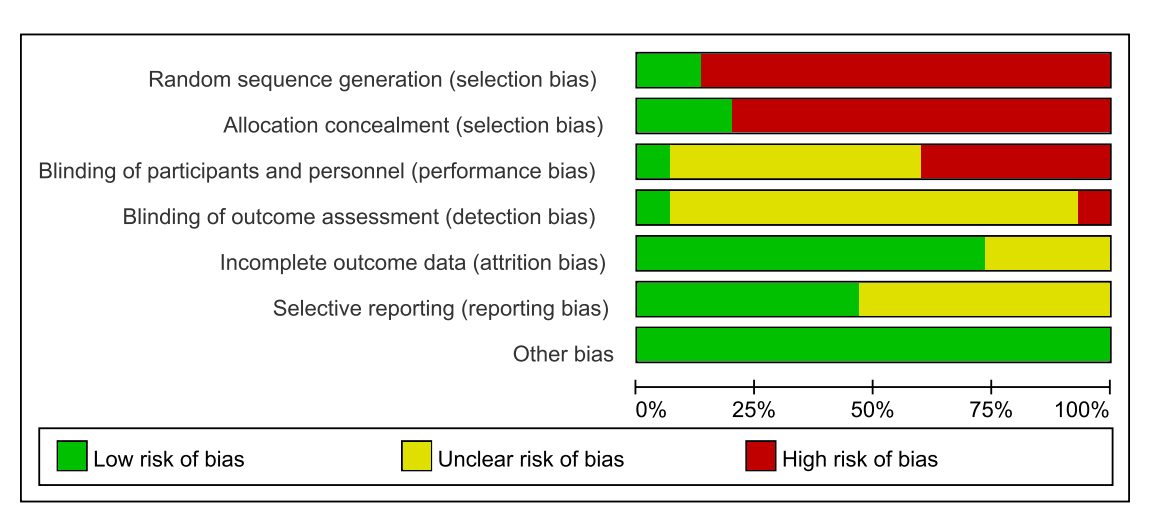
**Figure S1**. Risk of bias summary of included studies using Revman.

**Figure S2.** Brian regions with decreased signals compared CP and HCs.


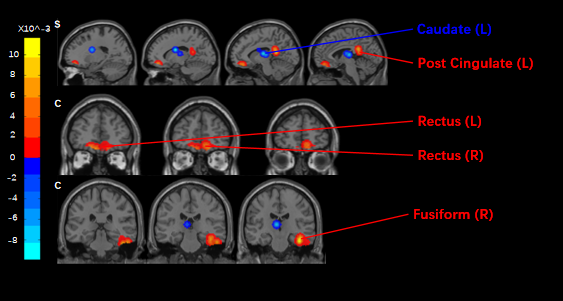


**Figure S3.** Brian regions with increased signals compared CP and HCs.


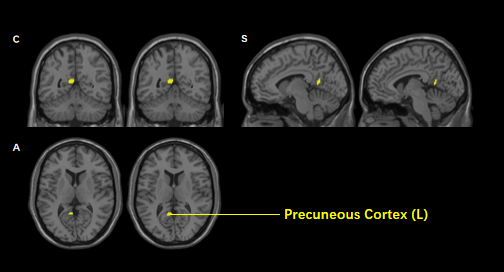


**Reporting quality control checklist**

0/0.5/1 score for each item; Totally 10 points. Give 0.5 points if some of the criteria are met.

**Category I:** subject quality

1. Patients were prospectively evaluated, specific diagnostic criteria were applied, and demographic data were reported.

2. Prospective evaluation of health comparison participants was performed to exclude mental and medical diseases.

3. Important variables (such as age, sex, course of disease, onset, medication, comorbidity, severity of disease) were stratified or statistically examined.

4. The sample size of each group is more than 10 cases.

**Category II:** research methods, image acquisition and analysis quality

5. Whole brain analysis was automated without prior regional selection.

6. Report coordinates in standard space.

7. The imaging technique used is clearly described so that it can be replicated.

8. The analytical method is clearly described so that it can be replicated.

**Category III:** quality of results and conclusions

9. Statistical parameters of significant differences and important non-significant differences are provided.

10. The conclusion is consistent with the obtained results, and the limitations are discussed.

| Source | Q1 | Q2 | Q3 | Q4 | Q5 | Q6 | Q7 | Q8 | Q9 | Q10 | Completeness  score |
| --- | --- | --- | --- | --- | --- | --- | --- | --- | --- | --- | --- |
| Zhao L, et al. (2014) | 1 | 0 | 0.5 | 1 | 1 | 1 | 1 | 1 | 0.5 | 1 | 8 |
| Hou XY, et al. (2014) | 1 | 0.5 | 0.5 | 1 | 1 | 1 | 1 | 0.5 | 0.5 | 1 | 8 |
| Chen WC, et al. (2015) | 1 | 1 | 0.5 | 1 | 1 | 1 | 1 | 0.5 | 0.5 | 1 | 8.5 |
| Han X, et al. (2017) | 0.5 | 0 | 0 | 0 | 1 | 1 | 1 | 1 | 0.5 | 1 | 6 |
| Zhang S, et al. (2018) | 0.5 | 0 | 0.5 | 0 | 1 | 1 | 1 | 1 | 0.5 | 1 | 6.5 |
| Makary, et al. (2018) | 0.5 | 0 | 0 | 1 | 1 | 1 | 1 | 1 | 0.5 | 1 | 7 |
| Zou Y, et al.  (2019) | 1 | 1 | 0.5 | 1 | 1 | 1 | 1 | 1 | 0.5 | 1 | 9 |
| Liu CH, et al. (2019) | 1 | 0.5 | 0.5 | 1 | 1 | 1 | 1 | 1 | 0.5 | 1 | 8.5 |
| Ning YZ, et al. (2020) | 1 | 1 | 0.5 | 1 | 1 | 1 | 1 | 1 | 0.5 | 1 | 9 |
| Li ZJ, et al.  (2020) | 1 | 0.5 | 0.5 | 1 | 1 | 1 | 1 | 1 | 0.5 | 1 | 8.5 |
| Zhang YT, et al. (2021) | 1 | 0 | 0.5 | 1 | 1 | 1 | 1 | 0.5 | 0.5 | 1 | 7.5 |
| Qu B, et al.  (2021) | 0.5 | 0 | 0.5 | 1 | 1 | 1 | 1 | 0.5 | 0.5 | 1 | 7 |
| Liu SS, et al. (2021) | 1 | 0.5 | 0.5 | 1 | 1 | 1 | 1 | 1 | 0.5 | 1 | 8.5 |
| Jia JN, et al.  (2021) | 1 | 0 | 1 | 1 | 1 | 1 | 1 | 0.5 | 0.5 | 1 | 8 |

**Table S1.** Study quality control checklist of included studies.

**Table S2.** The results of peak coordinates from ALE analysis comparing CP and HC.

| Cluster # | x | y | z | ALE | P | Z | Label (Nearest Gray Matter within 5mm) |
| --- | --- | --- | --- | --- | --- | --- | --- |
| 1 | 38 | -20 | -24 | 0.011688294 | 0.000 | 4.396 | Right Cerebrum.Temporal Lobe.Fusiform Gyrus.Gray Matter.Brodmann area 20 |
| 1 | 50 | -26 | -26 | 0.008020796 | 0.000 | 3.345 | Right Cerebrum.Temporal Lobe.Fusiform Gyrus.Gray Matter.Brodmann area 20 |
| 1 | 42 | -28 | -30 | 0.007834187 | 0.000 | 3.314 | Right Cerebrum.Temporal Lobe.Fusiform Gyrus.Gray Matter.Brodmann area 20 |
| 2 | -10 | 46 | -17 | 0.006446497 | 0.002 | 2.961 | Left Cerebrum.Frontal Lobe.Superior Frontal Gyrus.Gray Matter.Brodmann area 11 |
| 2 | -6.2 | 46 | -16.2 | 0.004137743 | 0.006 | 2.532 | Left Cerebrum.Frontal Lobe.Medial Frontal Gyrus.Gray Matter.Brodmann area 11 |
| 2 | 7 | 53 | -11 | 0.006098931 | 0.002 | 2.859 | Right Cerebrum.Frontal Lobe.Medial Frontal Gyrus.Gray Matter.Brodmann area 11 |
| 2 | -8 | 40 | -22 | 0.004496438 | 0.005 | 2.573 | Left Cerebrum.Frontal Lobe.Rectal Gyrus.Gray Matter.Brodmann area 11 |
| 3 | -12 | -44 | 16 | 0.010249834 | 0.000 | 4.118 | Left Cerebrum.Limbic Lobe.Posterior Cingulate.Gray Matter.Brodmann area 29 |
| 3 | -10 | -42 | 24 | 0.008871996 | 0.000 | 3.615 | Left Cerebrum.Limbic Lobe.Cingulate Gyrus.Gray Matter.Brodmann area 31 |
| 4 | -18 | 0 | 22 | 0.009955756 | 0.000 | -3.904 | Left Cerebrum.Sub-lobar.Caudate.Gray Matter.Caudate Body |
| 4 | -12 | -12 | 12 | 0.00885636 | 0.000 | -3.629 | Left Cerebrum.Sub-lobar.Thalamus.Gray Matter.Ventral Lateral Nucleus |
| 4 | -8 | -18 | 9 | 0.008280277 | 0.000 | -3.350 | Left Cerebrum.Sub-lobar.Thalamus.Gray Matter.Medial Dorsal Nucleus |

**Note:** A positive Z value means CP>HC; a negative Z value means CP＜HC.

**Abbreviations:** CP, chronic pain; HC, health control.
